# Reliability of a pressure pain threshold protocol: secondary analysis of a longitudinal trial with cluster randomization

**DOI:** 10.7717/peerj.20834

**Published:** 2026-02-25

**Authors:** Pedro Aguila-Humeres, Jaime Leppe-Zamora

**Affiliations:** School of Physiotherapy, Facultad de Medicina Clínica Alemana, Universidad del Desarrollo, Santiago, Chile

**Keywords:** Quantitative sensory testing, Pressure pain threshold, Reliability, Pain measurement, Protocol

## Abstract

**Background:**

Pressure Pain Threshold (PPT) measurement is a useful method for assessing pain sensitivity when applied with a standardized protocol. However, little is known about the longitudinal stability of reliability estimates when PPT protocols are embedded in randomized controlled trials. This study aimed to estimate the relative and absolute reliability of a PPT algometry protocol in office workers across multiple time points within a six-month pragmatic randomized controlled trial.

**Methods:**

A secondary analysis was conducted from a cluster randomized controlled trial with office workers, where a standardized PPT protocol was applied to the neck, forearm, and lower leg. Measurements were taken bilaterally with three repetitions per site. Measurement reliability was assessed with a two-way mixed-effects intraclass correlation (ICC) model for absolute agreement (3, k), along with the Standard Error of Measurement (SEM) and Minimal Detectable Change (MDC), calculated at baseline, three, and six months, for both intervention and control groups.

**Results:**

ICC values ranged from 0.84 to 0.95, indicating good to excellent reliability across all time points and body regions. SEM ranged from 0.23 kg/cm^2^ to 0.51 kg/cm^2^, and MDC from 0.52 kg/cm^2^ to 1.15 kg/cm^2^. These values remained consistent across follow-up periods in both groups, despite expected variability due to the intervention.

**Conclusions:**

The PPT protocol demonstrated high measurement reliability and stable properties over time for each anatomic region and both groups, supporting its use in longitudinal assessments of pain sensitivity in occupational and clinical settings.

## Introduction

Pain evaluation remains a challenge in clinical and research settings because of its complexity as a construct based on self-experience. Pain is defined as “an unpleasant sensory and emotional experience associated with, or resembling that associated with, actual or potential tissue damage” (Raja et al., 2021). This subjective experience has been associated with three main elements to explain the expression of pain, such as cognitive (Khera & Rangasamy, 2021), affective (Elman & Borsook, 2018), and sensory components of the nervous system (Garland, 2012). The complexity of the painful experience lies in the capacity of the nervous system to associate these components, allowing pain to function as an adaptive response (Moseley & Butler, 2015).

Although scales and questionnaires are the most commonly used tools to measure pain outcomes (Crisman et al., 2024; Karcioglu, Topacoglu & Dikme, 2018; Jayaseelan, Scalzitti & Courtney, 2023), one set of tools that have demonstrated promising potential in assessing pain mechanisms are the quantitative sensory tests (QST) (Shraim et al., 2022; Hodges et al., 2025). These standardized procedures rely on the assessment of the somatosensory pain function, eliciting responses to touch, vibration, pinpricks, pressure, or thermal stimuli (Suzuki et al., 2022). Because the QST measures an objective outcome (*e.g.*, pressure) associated with a subjective response (*e.g.*, pain), it can provide a valuable opportunity to explore pain mechanisms and pain experience using a quantitative continuous ratio variable (Hodges et al., 2025).

Within the QST measurements, pressure pain threshold (PPT) refers to the detection of pain as a result of pressure applied on a given part of the body (Suzuki et al., 2022). This method of quantifying pain sensitivity reflects the processes of nociceptive transduction and peripheral transmission, whereby input from pressure stimuli is encoded and interpreted as pain (Suzuki et al., 2022). Moreover, contemporary pain literature conceptualizes pain as a complex, biopsychosocial phenomenon involving both sensory and emotional experience (Raja et al., 2021). Therefore, the resulting sensation captured by PPT is not solely a product of peripheral nociceptor activation but reflects the integration of peripheral input with extensive central nervous system processing (Geri et al., 2022; Garland, 2012). This experience is significantly modulated by psychological factors, including affective and cognitive components of the nervous system (Khera & Rangasamy, 2021; Elman & Borsook, 2018; Garland, 2012). Consequently, the variability observed in PPT measurements is highly dependent upon and related to individual characteristics, which actively contribute to pain perception (Georgopoulos et al., 2019; El-Sayed et al., 2021; Vervullens et al., 2022; Steinmetz, Hacke & Delank, 2023).

Evidence suggests that PPT may be responsive to change and can reflect patient-perceived improvement in neck pain. In an anchor-based analysis, upper trapezius PPT demonstrated moderate discrimination between patients who achieved clinically meaningful improvement and those who did not, and it showed comparatively higher specificity for ruling out change when thresholds remain stable (Walton et al., 2014).

One type of intervention that has shown small to moderate effects on modifying the PPT is the increase in physical activity through structured exercises. Increases in PPT associated with such interventions have been linked to resistance training targeting specific muscle groups, as well as aerobic exercise, with evidence supporting their effectiveness in both healthy populations and individuals with musculoskeletal disorders (Heredia-Rizo et al., 2019; Pacheco-Barrios et al., 2020; Ylinen et al., 2005; Burrows et al., 2014; Leźnicka et al., 2022; Lee, 2014). Exercise-induced alterations in PPT exhibit mixed durations, with acute, transient increases following a single bout that typically resolve within-session (Pacheco-Barrios et al., 2020; Burrows et al., 2014; Lee, 2014), in contrast to longer-term programs that produce sustained improvements over weeks to months (Heredia-Rizo et al., 2019; Ylinen et al., 2005; Leźnicka et al., 2022).

While PPT measurement is potentially valuable for assessing changes over time due to interventions such as the use of standardized exercises, to date, little is known about the longitudinal stability of reliability estimates when PPT protocols are embedded in randomized controlled trials (Bhattacharyya et al., 2023). In this context, it is essential to evaluate how reliability parameters evolve over time, ensuring that observed intervention effects can be distinguished from measurement error rather than relying solely on baseline estimates. To ensure methodological rigor, such evaluations should incorporate both relative and absolute reliability, expressed through the intraclass correlation coefficient (ICC), the standard error of measurement (SEM), and the minimal detectable change (MDC), as these indices capture the capacity to distinguish true change from measurement error (Mokkink et al., 2020). Building on prior evidence showing that digital algometry provides high intra- and inter-rater reliability (Bhattacharyya et al., 2023; Straatman et al., 2023; Waller et al., 2015) and concurrent validity (Castien et al., 2021), the present study offers an opportunity to examine how reliability parameters change over time within a longitudinal design.

Therefore, this study aimed to estimate the relative and absolute reliability of a PPT algometry protocol in office workers across multiple time points within a six-month pragmatic randomized controlled trial, including site-specific estimates of ICC, SEM, and MDC.

## Materials & Methods

### Study design

This study presents a secondary analysis of a two-arm parallel pragmatic randomized controlled trial, with assessments at baseline and at 3 and 6 months (ClinicalTrials.gov ID: NCT05790837). In the main study, the active group received an intervention aimed to reduce sedentary behavior and increase physical activity levels through the “Ponte de Pie” (Stand-up), a computer prompt developed by the Chilean Health Ministry. This software, installed on participants’ work computers, triggered a two-minute standardized video every 60 min of continuous computer work, encouraging standing exercises, stretching, and deep breathing. Meanwhile, the control group maintained their usual work routines and received only written recommendations on reducing sedentary behavior.

### Participants

Office workers from two university institutions in Santiago, Chile were invited to participate in the main study. A sample of office workers was included due to the high prevalence of musculoskeletal disorders in this population, particularly in the neck and upper limbs (Jun et al., 2017; Nakatsuka et al., 2021; Cagnie et al., 2007; Eltayeb et al., 2007; Bretschneider et al., 2022), where such complaints have been associated with prolonged sitting and insufficient physical activity (Dzakpasu et al., 2021; Putsa et al., 2022; Parry et al., 2019). Moreover, studies have shown that variations in physical activity and sedentary behavior are associated with the prevalence of musculoskeletal pain in occupational contexts (Leppe Zamora et al., 2025), underscoring the importance of preventive strategies in this population (Parry et al., 2019; Leppe Zamora et al., 2025). Consequently, studying office workers provides a valuable opportunity to evaluate changes in pain perception, both as a preventive initiative and as a treatment approach, from an occupational health perspective with potential applicability to clinical practice.

The recruitment period took place between August and December of 2023, and data collection was conducted between September 2023 and June 2024. Participants were eligible if they were over 18 years old, spent more than 60% of their working day sitting, were a full-time employee (working more than 35 h per week) and could walk independently without assistive devices or requiring assistance from another person. Exclusion criteria included pregnancy, the use of height-adjustable workstation, and meeting the WHO criteria for sufficient activity (World Health Organization, 2020), which was self-reported by the participants at the moment of recruitment. All participants provided written informed consent before data collection, in accordance with the approval act No. 2023-29 of the Scientific Ethics Committee at Universidad del Desarrollo.

### Outcome of interest

#### Pain sensitivity

The measure of pain sensitivity was conducted through a PPT protocol developed according to the characteristics of the study design (pragmatic randomized trial) (Zuidgeest et al., 2017), where data collection took place in a real-world setting within the participants’ work environment. PPT was measured by a steady rate of pressure application perpendicular to the body, and participants were instructed to indicate when a pain sensation first appeared. The result of this test is expressed in a force-pressure value (kg/cm^2^).

### Musculoskeletal pain prevalence

The main study assessed musculoskeletal pain with the Standardised Nordic Questionnaire (Kuorinka et al., 1987; Martínez & Alvarado Muñoz, 2017) before PPT testing. The instrument records three dichotomous items (yes/no) for each body region—pain in the past 12 months, interference with normal work, and pain in the past 7 days. Although these data were not used for eligibility and were not part of the reliability analyses, their descriptive results are reported in the Results section.

### PPT protocol

#### Assessment setting

Assessments were conducted in laboratories within the participating universities where the workers were employed, simulating a clinical environment. The setting was standardized for all time points measurements—(controlled lighting and noise, stable room temperature, uniform examination surfaces, and privacy).

### Device

A handheld digital algometer (FPX^®^-25 series, Wagner Instruments, Greenwich, USA) with a one cm^2^ round rubber tip and a linear range of 0–14 kg/cm^2^ was used. The device operated in compression mode to capture applied force and in peak mode to hold the maximum value for recording.

### Participant instructions and familiarization

Before testing, raters delivered a standardized script instructing participants to indicate the first moment when the sensation changed from pressure to an unpleasant/painful feeling, consistent with the IASP definition (Raja et al., 2021). If needed, “unpleasant” was clarified as the initial indication of pain. A brief demonstration was performed at the first interdigital space of the right hand while seated to confirm understanding and familiarize the participant with the sensation.

### Positioning and sites

Three regions were assessed bilaterally using standard landmarks. The neck/upper trapezius (identify C7 and measure the midpoint toward the acromion along a straight line), the elbow/extensor carpi ulnaris (locate the lateral epicondyle and mark the muscle belly 5–6 cm distal, confirm by resisted finger extension), and the lower leg/tibialis anterior (palpate the fibular head and mark the muscle belly 5–6 cm distal, confirm by dorsiflexion). In the main study, the neck and forearm sites were chosen according to office-worker pain-prevalence data (Jun et al., 2017; Nakatsuka et al., 2021; Cagnie et al., 2007), while the lower leg site was included as a distal comparator in line with published PPT protocols (Walton et al., 2014; Nunes & Leite, 2025), given that concurrent alterations at local and distal locations can indicate clinically widespread mechanical hyperalgesia (Hodges et al., 2025; Nunes & Leite, 2025).

All measurements were obtained with the participant supine on an examination table, palms facing down. This positioning aligns with prior PPT reliability protocols for the same regions (Trueba-Perdomo, Gasparini & Flores Cuautle, 2021; Knapstad et al., 2018; Jones, Kilgour & Comtois, 2007; Nunes & Leite, 2025) and was selected to minimized duration of the protocol by avoiding repositioning, given the pragmatic characteristics of the main study.

### Measurement sequence and rate

Measurements proceeded from neck to forearm and finally the lower leg, alternating right and left sides. At each site, three repetitions were recorded to capture within-site variability, consistent with recommendations (Balaguier, Madeleine & Vuillerme, 2016; Konrad et al., 2023). Pressure was applied at a steady rate of 1 kg/cm^2^/s, monitored on the algometer display. A 30-s inter-trial interval was used in line with similar reliability protocols (Ylinen et al., 2005; Trueba-Perdomo, Gasparini & Flores Cuautle, 2021; Knapstad et al., 2018; Nunes & Leite, 2025) and given the limited time available to implement the protocol during the participants’ working day. The full protocol required about 15 min per participant.

### Raters and training

Two trained physiotherapists administered the protocol across all time points, where each participant was assessed by the same rater within a session on a given time point. However, because the trial’s primary objective was not reliability testing, a different rater could assess the same participant at subsequent time points (at study end, 60% of participants were reassessed by the same rater and 40% by a different rater). Prior to data collection, raters completed structured practice with each other to familiarize themselves with the device, the pressure-application rate, anatomical site identification, and the standardized script, thereby ensuring consistency. The raters were not blinded to the measurement values shown on the device screen or to the participants’ group assignment.

### Materials

Detailed checklists for verbal instructions, positioning, landmarks, rate control, and the recording sheet are provided in File S1.

### Statistical analysis

The data distribution was analyzed using the Shapiro–Wilk normality test. Descriptive statistics were reported as mean and standard deviation (SD) for normally distributed data and as median with interquartile range (IQR) for non-parametric distributions.

For each anatomical region (neck, forearm, and lower leg), the three repeated measurements per side were averaged. As no significant differences were found between contralateral sides, the final PPT value per region was calculated as the mean of both sides.

Measurement reliability of the protocol was assessed with a within–time-point approach, using data from each participant’s session at baseline, 3 months, and 6 months. For each group, we estimated relative reliability (ICC) and absolute reliability (SEM, MDC) from the repeated measurements collected in that time point. An available-cases strategy was applied, including only participants with complete PPT repeats at the corresponding time point.

For relative reliability, the intraclass correlation coefficient (ICC) was calculated for each region with a two-way mixed-effects model for absolute agreement (Model 3, k) (Koo & Li, 2016). This model is considered appropriate for reliability studies utilizing the mean of repeated (*k*) measurements, and when the selected sample of raters is the only set of interest (Koo & Li, 2016). Given the objective of stablish the measurement reliability of the protocol, an absolute agreement was selected because it evaluates numerical equivalence across repeated measurements, incorporating both association and systematic differences rather than correlation alone. This approach provides a reliable estimate by accounting for within-subject variance and including the effect of systematic measurement bias (Koo & Li, 2016; Liljequist, Elfving & Skavberg Roaldsen, 2019; Mokkink et al., 2023). ICC values were interpreted according to the following criteria: <0.5 (poor), 0.51–0.75 (moderate), 0.75–0.90 (good), and >0.9 (excellent) (Portney, 2020).

Absolute reliability was determined using the standard error of measurement (SEM), calculated as SEM = SD ×$\sqrt{(1-\mathrm{ICC})}$ (Weir, 2005). The SD used for this calculation was obtained from the distribution of the final averaged PPT values per body region, which were constructed from the three repeated measurements on each side. Because the SD used for the SEM must derive from the same dataset as the ICC estimate, separate SD values were calculated for each time point (Weir, 2005; De Vet et al., 2011).

Additionally, the minimal detectable change (MDC) was calculated at a 90% confidence level using the formula: MDC = SEM × $\sqrt{2}$ ×1.64 (Mailloux et al., 2021). Conceptually, the MDC reflects a statistical threshold of change derived from measurement error and depends on the level of variability of the measurement, as captured by the ICC and the SEM (Weir, 2005).

### No imputation was performed

All analyses were performed using R software (version 4.3.3; R Core Team, 2024).

## Results

**Figure 1:**
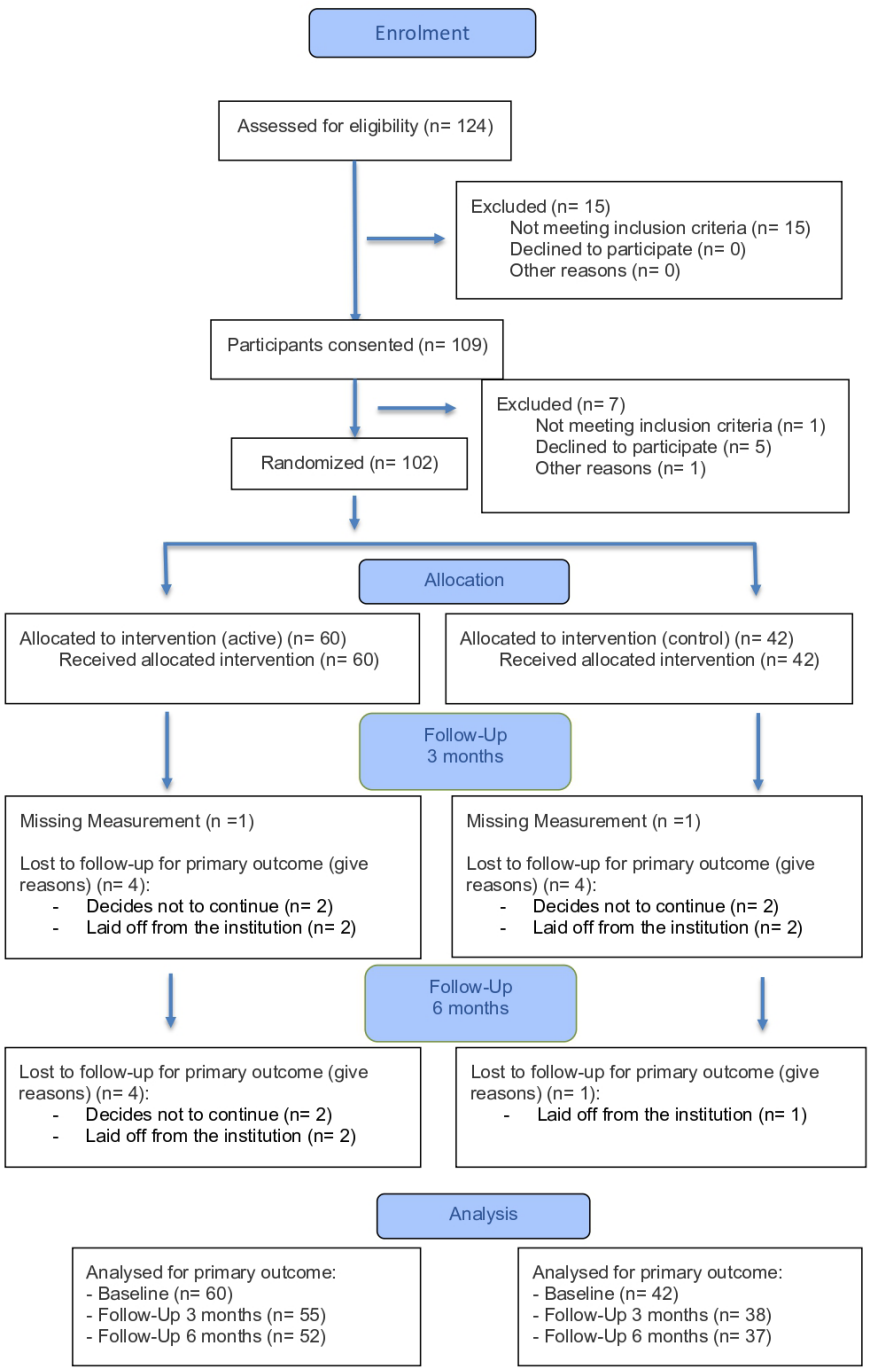
Flow-chart of the PPT measurements. CONSORT flow diagram of participant progression through the study. A total of 124 office workers were assessed for eligibility, of which 109 provided informed consent. After applying exclusion criteria, 102 participants were randomized into two groups: intervention (*n* = 60) and control (*n* = 42). All participants received their allocated intervention. Follow-up at 3 and 6 months included minor loss to follow-up in both groups, mainly due to participants’ withdrawal or institutional job termination. Final analysis was performed on all randomized participants in accordance with the intention-to-treat principle.

Of the 124 participants assessed for eligibility, 109 signed the consent document and 102 participants were randomized, with 60 participants allocated in the active group and 42 in the control group. Considering the available-cases analysis at each time point, in the active group, *n* = 60 were analyzed at baseline, *n* = 55 at 3 months, and *n* = 52 at 6 months, while in the control group, *n* = 42, *n* = 38, and *n* = 37 were analyzed at the respective time points (Fig. 1). The sample consisted of 85% women, with a mean age of 45 years (±11.2) and a median BMI of 27.8 (IQR: 25.1–31.2). On average, participants spent 80% of their working time sitting, and had a prevalence of 46% of neck pain and 19% of elbow pain within the last seven days. Baseline characteristics are reported in Table 1.

**Table 1:** Descriptive characteristics of the study participants at baseline. Each data point represents baseline descriptive characteristics of the study participants (*N* = 102), stratified by intervention group (Active Group, *n* = 60; Control Group, *n* = 42). Continuous variables are presented as mean ± standard deviation or median with interquartile range (IQR), depending on distribution. Categorical variables are presented as frequencies and percentages. OSPAQ responses describe the self-reported percentage of time spent sitting, standing, and walking during work hours. Occupational classifications follow the ISCO-08 framework. No significant differences were observed between groups at baseline.

	Total (*n* = 102)	Active group (*n* = 60)	Control group (*n* = 42)
Age (years)	44.9 ± 11.8	44.7 ± 7.5	47.9 ± 10.98
Sex, female (%)	89 (87%)	48 (80%)	41 (98%)
Body mass index (kg/m^2^)	27.8 (25.1–31.2)	28.2 (25.7–31.8)	27.2 (24.4–30.2)
OSPAQ (%, IQR)			
Sitting	80% (80–90)	80% (80–90)	80% (80–90)
Standing	5% (0–10)	5% (0–10)	0% (0–5)
Walking	10% (5–19)	10% (5–15)	10% (8–20)
Nordic questionnaire (%)			
Neck pain within last 12 months	77.5	81.7	71.4
Neck pain interference in normal work performance within last 12 months	32.4	46.7	11.9
Neck pain within last 7 days	46.1	46.7	45.2
Elbow pain within last 12 months	27.5	30	23.8
Elbow pain interference in normal work performance within last 12 months	13.7	16.7	9.5
Elbow pain within last 7 days	18.6	21.7	14.7
Working hours/week (n, %)			
40–44	17 (17%)	15 (25%)	2 (5%)
45	85 (83%)	45 (75%)	40 (95%)
Major occupational groups (n, %)			
Managers	1 (1%)	1 (2%)	0 (0%)
Professionals	45 (44%)	25 (42%)	20 (48%)
Technicians and associated professionals	26 (26%)	15 (25%)	11 (26%)
Clerical support workers	30 (29%)	19 (32%)	11 (26%)

**Notes.**

Body Mass Index: kg/m^2^. OSPAQ: Occupational Sitting Physical activity. Major Occupational Groups classified according to ISCO-08. Mean (standard deviation), Median with % or IQR (Interquartile range p25–p75) or Frequency (%).

### PPT measurements

In the overall sample, baseline PPT values were similar between neck and forearm, while the lower leg showed higher values, with a mean of 2.52 ±1.0 kg/cm^2^ on the neck, 2.71 ± 1.10 kg/cm^2^ on the elbow and 4.12 ± 1.70 kg/cm^2^ on the lower leg. Over follow-up, the active group showed increases at 3 months with partial retention at 6 months, particularly at the neck and the lower leg, whereas PPT at the forearm tended to be stable. In contrast, the control group tended toward stability or slight decline in the three sites measured, with decreases at the lower leg and the neck, and smaller fluctuations at the forearm. Results across groups, baseline and follow-up periods are provided in Table 2.

**Table 2:** Pressure pain threshold values at baseline and follow-up in neck, forearm, and reference regions, stratified by intervention group. Each data point represents the mean pressure pain threshold (PPT) in kg/cm^2^ ± standard deviation for three anatomical regions (neck, forearm, and a reference site), assessed at baseline, 3 months, and 6 months. Results are shown separately for the Active and Control groups. PPT values reflect sensitivity to mechanical pressure, with higher scores indicating greater tolerance. The reference site represents a body region with minimal exposure to occupational load

Body Region	Total baseline (kg/cm^2^) (*n* = 102)	PPT by group	Baseline PPT (kg/cm^2^) Active (*n* = 60) Control (*n* = 42)	3 Months PPT (kg/cm^2^) Active (*n* = 55) Control (*n* = 38)	6 Months PPT (kg/cm^2^) Active (*n* = 52) Control (*n* = 37)
Neck	2.52 ± 1.0 (2.32–2.72)	Active	2.33 ± 0.95 (2.09–2.57)	2.96 ± 0.96 (2.70–3.21)	2.71 ± 0.81 (2.48–2.93)
		Control	2.79 ± 1.06 (2.46–3.12)	2.74 ± 0.93 (2.43–3.04)	2.61 ± 0.94 (2.29–2.92)
Forearm	2.71 ± 1.10 (2.50–2.92)	Active	2.63 ± 0.99 (2.37–2.88)	2.75 ± 0.88 (2.52–2.97)	2.71 ± 0.79 (2.50–2.91)
		Control	2.84 ± 1.18 (2.47–3.20)	2.49 ± 0.83 (2.22–2.76)	2.63 ± 0.83 (2.35–2.91)
Lower leg	4.12 ± 1.70 (3.80–4.45)	Active	3.84 ± 1.44 (3.47–4.21)	4.31 ± 1.56 (3.90–4.74)	4.19 ± 1.37 (3.81–4.58)
		Control	4.52 ± 1.91 (3.92–5.12)	4.07 ± 1.53 (3.57–4.58)	3.95 ± 1.59 (3.42–4.48)

### Reliability

The relative reliability of the PPT protocol showed ICC values ranging from good to excellent for the total sample and both groups across all time points. At baseline, the ICC was 0.92 (CI [0.88–0.95]) for the neck, 0.91 (CI [0.87–0.94]) for the forearm, and 0.92 (CI [0.86–0.95]) for the lower leg, considering all participants. The SEM at baseline was 0.29 kg/cm^2^ for the neck, 0.32 kg/cm^2^ for the forearm, and 0.48 kg/cm^2^ for the lower leg. The MDC values were 0.67 kg/cm^2^ for the neck, 0.73 kg/cm^2^ for the forearm, and 1.1 kg/cm^2^ for the lower leg.

In the control group, the ICC ranged from 0.91 to 0.95 across all body regions from baseline to follow-up, with a SEM that went from 0.24 kg/cm^2^ to 0.25 kg/cm^2^ on the neck, 0.22 kg/cm^2^ to 0.35 kg/cm^2^ on the forearm and 0.36 kg/cm^2^ to 0.50 kg/cm^2^ on the lower leg from baseline to follow-up. The MDC ranged from 0.56 kg/cm^2^ to 0.59 kg/cm^2^ on the neck, 0.52 kg/cm^2^ to 0.81 kg/cm^2^ on the forearm and 0.83 kg/cm^2^ to 1.15 kg/cm^2^ on the lower leg, from baseline to follow-up.

In the active group, the lowest ICC was 0.85 while the highest was 0.93 across all measurements. The SEM varied from 0.29 kg/cm^2^ to 0.37 kg/cm^2^ on the neck, 0.24 kg/cm^2^ to 0.29 kg/cm^2^ on the forearm, and 0.42 kg/cm^2^ to 0.51 kg/cm^2^ on the lower leg, with an MDC that ranged from 0.67 kg/cm^2^ to 0.85 kg/cm^2^ for the neck, 0.56 kg/cm^2^ to 0.67 kg/cm^2^ for the forearm, and 0.94 kg/cm^2^ to 1.19 kg/cm^2^ for the lower leg throughout the study.

All the reliability results with the ICC values and their respective CI as well as the SEM and the MDC for both groups are shown in Table 3.

**Table 3:** Relative and absolute reliability of pressure pain threshold measurements across time points and body regions, by intervention group. Each data point represents reliability metrics for pressure pain threshold (PPT) measurements at baseline, 3 months, and 6 months, stratified by intervention group (Active and Control) and body region (neck, forearm, and reference). Relative reliability is reported as the intraclass correlation coefficient (ICC) with 95% confidence intervals, while absolute reliability is indicated by the standard error of measurement (SEM) and the minimal detectable change (MDC).

		Baseline Total (*n* = 102) Active (*n* = 60) Control (*n* = 42)			3 Months Active (*n* = 55) Control (*n* = 38)			6 Months Active (*n* = 52) Control (*n* = 37)		
Body Region	Group	ICC (95% CI)	SEM (kg/cm^2^)	MDC (kg/cm^2^)	ICC (95% CI)	SEM (kg/cm^2^)	MDC (kg/cm^2^)	ICC (95% CI)	SEM (kg/cm^2^)	MDC (kg/cm^2^)
Neck	Active	0.90 (0.82–0.94)	0.29	0.67	0.85 (0.74–0.91)	0.37	0.85	0.84 (0.73–0.91)	0.32	0.74
	Control	0.94 (0.89–0.97)	0.25	0.59	0.93 (0.88–0.96)	0.24	0.56	0.93 (0.87–0.97)	0.24	0.56
	Total	0.92 (0.88–0.95)	0.28	0.64	–	–	–	–	–	–
Forearm	Active	0.91 (0.86–0.95)	0.29	0.67	0.90 (0.83–0.94)	0.28	0.65	0.91 (0.83–0.95)	0.24	0.56
	Control	0.91 (0.84–0.95)	0.35	0.81	0.92 (0.86–0.96)	0.23	0.53	0.93 (0.85–0.96)	0.22	0.52
	Total	0.91 (0.87–0.94)	0.32	0.73	–	–	–	–	–	–
Lower leg	Active	0.90 (0.78–0.95)	0.42	0.97	0.93 (0.87–0.96)	0.42	0.98	0.86 (0.76–0.92)	0.51	1.19
	Control	0.93 (0.87–0.96)	0.50	1.15	0.92 (0.84–0.96)	0.44	1.03	0.95 (0.90–0.97)	0.36	0.83
	Total	0.92 (0.86–0.95)	0.48	1.10	–	–	–	–	–	–

**Notes.**

ICC Intraclass Correlation Coefficient SEM Standard Error Measurement MDC Minimal Detectable Change (90%)

## Discussion

This study examined the measurement reliability of a standardized PPT protocol in office workers over a six-month pragmatic randomized controlled trial. The results demonstrated a good to excellent relative reliability, with ICC values ranging from 0.86 to 0.95 across all regions and time points. These findings support the reproducibility and consistency of the protocol for evaluating pain sensitivity in this population. Baseline ICC values exceeded 0.90 across all body regions, consistent with previous findings using digital algometry (Bhattacharyya et al., 2023; Trueba-Perdomo, Gasparini & Flores Cuautle, 2021; Bellosta-López et al., 2023). At three- and six-month follow-ups, a slight reduction in ICC was observed in the active group, particularly for the neck and lower leg. Rather than indicating a limitation of the protocol, this decrease more likely reflects inter-individual differences in response to the intervention. In longitudinal studies, such variability is expected and can increase the error term, thereby influencing ICC values. Importantly, in this context, the observed increase in error appears to reflect meaningful variability in the construct being measured—pain sensitivity—driven by the intervention itself, rather than random measurement noise. This distinction is crucial in designs that include both intervention and control groups, as changes in variability may represent true biological responses rather than measurement flaws (Weir, 2005).

A key aspect in interpreting these findings is the role of SEM and MDC in evaluating reliability within longitudinal designs (Mokkink et al., 2020; Mokkink et al., 2023). Traditionally, SEM estimates the degree of measurement error in a single assessment, while MDC provides a threshold for distinguishing real changes from random measurement variability (Trueba-Perdomo, Gasparini & Flores Cuautle, 2021; Balaguier, Madeleine & Vuillerme, 2016). However, both metrics are sensitive to inter-individual variability in response to intervention in follow-up assessments (Weir, 2005). In our study, the observed changes in SEM and MDC likely reflect actual variation in pain sensitivity among participants rather than inconsistency in the measurement tool. This interpretation is reinforced by the control group, in which ICC values remained stable (0.91–0.95) across regions and time points and absolute reliability indices (SEM/MDC) were broadly consistent over time, demonstrating the robustness of the PPT protocol. When intervention elicit heterogeneous responses, SEM increases, leading to a higher MDC and reduced sensitivity to subtle changes. In contrast, homogeneous responses may lower SEM, improving measurement precision (DeVet et al., 2006). For this reason, SEM and MDC should be recalculated at each time point to account for evolving response variability. This approach allows for a more accurate change interpretation by differentiating between intervention effects and measurement errors.

Knowing the MDC is particularly relevant when evaluating interventions that use PPT as an outcome measure, as it establishes the smallest change that can be confidently distinguished from measurement error (Mokkink et al., 2020; Weir, 2005). In the active group, the MDC values varied across anatomical sites, with thresholds as high as 0.85 kg/cm^2^ for the neck, 0.67 kg/cm^2^ for the forearm, and 1.19 kg/cm^2^ for the lower leg, indicating site-specific differences in the sensitivity of PPT to detect true changes. These values provide clinicians and researchers with a benchmark to determine whether observed differences reflect genuine effects of an intervention rather than random variability, an essential consideration when interpreting treatment efficacy. Nevertheless, as highlighted by de Vet and colleagues, the MDC reflects statistical reliability rather than clinical significance, and should not be equated with the Minimally Important Difference (MID), which uniquely captures whether a change is meaningful from the patient’s perspective (DeVet et al., 2010; Dekker, De Boer & Ostelo, 2024).

The relative reliability estimator was the ICC calculated with a two-way mixed-effects for absolute agreement. This model was selected because the raters constituted a fixed set and our aim was numerical agreement of the averaged score across repeats (Koo & Li, 2016; Liljequist, Elfving & Skavberg Roaldsen, 2019). Under the absolute-agreement framework, systematic rater offsets are incorporated into the error term, which is appropriate when deriving absolute indices such as the SEM and the MDC from the observed-score variance and from the ICC for the average of k repeats (Koo & Li, 2016; Liljequist, Elfving & Skavberg Roaldsen, 2019; Mokkink et al., 2023). Nevertheless, the construct assessed by PPT also requires the protocol to preserve the rank ordering of between-subject differences. For this reason, a comparative approach is recommended in which both agreement and consistency are examined. A consistency ICC that is substantially higher than the absolute-agreement ICC indicates non-negligible systematic error, and reporting both coefficients provides complementary information about the reliability of the method (Liljequist, Elfving & Skavberg Roaldsen, 2019; Mokkink et al., 2023). In our data, the two definitions yielded similar values, which indicates no meaningful systematic bias (Liljequist, Elfving & Skavberg Roaldsen, 2019) (consistency-based results are provided in Supplemental Information 3).

The mean PPT values at baseline for all participants were 2.52 kg/cm^2^ (±1.0) for the upper trapezius, 2.71 kg/cm^2^ (±1.1) for the extensor carpi ulnaris and 4.12 kg/cm^2^ (±1.7) for the tibialis anterior. These results are consistent with those reported in the literature considering the body regions measured on office workers. The study of Ge et al. (2014) evaluated the PPT in computer users with and without chronic musculoskeletal pain, with a mean value of 2.64 kg/cm^2^ (±0.41) for the upper trapezius, 2.71 kg/cm^2^ (±0.19) on the extensor carpi ulnaris and 4.24 kg/cm^2^ (±1.69) on the tibialis anterior for the control group of healthy subjects. Similarly, the study of Heredia-Rizo et al. (2019) showed a PPT mean of 2.13 kg/cm^2^ (±0.83) for the upper trapezius and 2.84 kg/cm^2^ (±1.21) for the extensor carpi ulnaris at baseline for the control group of female computer users with chronic neck/shoulder pain. Interestingly, the PPT values between office workers with and without chronic pain seem to be similar without significant differences. The systematic review of Nunes et al. (2021) analyzed the PPT values among office workers with chronic neck pain compared to asymptomatic control groups. Considering only studies with above-average methodological quality, the meta-analysis for the upper trapezius showed that PPT among office workers with chronic neck pain was not significantly lower than in workers without neck pain (mean difference −10.89 kPa; 95% CI [−36.16–14.38]; I^2^ = 0%, *p* = 0.64; *χ*^2^ = 1.67). However, these findings should be interpreted with caution, as the discriminative ability of PPT may vary with the PPT protocol and the clinical status or pain severity of the population. Taken together, the small or non-significant between-group differences observed in office workers with *versus* without chronic neck pain, alongside the moderate ability of upper trapezius PPT to detect clinically meaningful improvement over time (Walton et al., 2014), suggest that upper trapezius PPT is suited to track within-person changes. Accordingly, upper trapezius PPT may be most informative for evaluating intervention response, particularly when interpreted alongside reliability indices (ICC, SEM, MDC) and pre-specified minimal important change thresholds.

### Limitations

This study presents certain limitations that should be considered when interpreting the findings. A key limitation is that this is a secondary analysis of a randomized clinical trial whose design and sample size were powered for intervention effects rather than for reliability hypotheses. Precision in ICC estimates depends on sample size, where small samples typically tend to produce unstable ICC estimates, leading to wider confidence intervals and reduced interpretability (Shieh, 2014; Mokkink et al., 2022). According to the simulation-based recommendations of Mokkink et al. for two-way mixed-effects models with three or more repeats, gains in precision plateau beyond approximately 40–50 participants, particularly when ICC exceeds 0.8 (Mokkink et al., 2022). In our data, ICCs of 0.86–0.95 with 95% confidence-interval widths of 0.07–0.18 are consistent with these expectations.

Although our results reflect high relative reliability and stable site-specific absolute reliability under a within–time-point stability of repeated measurements, in the main study design raters were not crossed with all participants across time points. This structure complicates the partitioning of variance components, and systematic rater effects cannot be fully separated from residual error. Nonetheless, the explicit methodological selection of an absolute agreement measure designed to account for all error sources (Koo & Li, 2016; Weir, 2005). Considering that consistency ICC substantially higher than the absolute-agreement ICC indicates non-negligible systematic error, reporting both coefficients provides complementary information about the reliability of the method (Liljequist, Elfving & Skavberg Roaldsen, 2019; Mokkink et al., 2023). In our data, the two definitions yielded similar values, which indicates no meaningful systematic bias (Liljequist, Elfving & Skavberg Roaldsen, 2019). Consistency-based results are provided in Supplemental Information 3. Future studies should use a fully crossed design to quantify intra-rater and inter-rater reliability to strengthen external validity.

Measurement reliability of the protocol was assessed using an available-cases analysis, based on repeated measurements obtained at each time point and including only participants evaluated during the corresponding period. Although this approach is appropriate for assessing the stability of reliability estimates of the PPT protocol across different time points, alternative analytic choices—such as a completers-only analysis (participants who completed all follow-ups)—may yield dissimilar estimates (Enders, 2004). To assess robustness, we report a completers-only sensitivity analysis in Supplemental Information 3.

Although the use of a convenience sample—arising from the decision to recruit participants exclusively from two universities—may introduce selection bias, the randomized design of the main trial remains a key strength in ensuring internal validity and controlling for confounding factors. Furthermore, the type of institution selected allowed for the inclusion of a diverse range of occupational roles, covering four International Standard Classification of Occupations (ISCO-08) major groups. The generalizability of the present findings is influenced by specific characteristics of the study design and participant selection. The main study was a pragmatic randomized trial conducted in an occupational context rather than a controlled clinical setting. While this approach introduces greater variability (“noise”) due to less control over environmental factors, it also enhances the applicability of the results to more controlled environments.

Because 85% of the sample was female and sex differences in PPT sensitivity are well documented, this imbalance may limit the generalizability of the reliability estimates. Given that ICC estimates rely on within-sample variability (DeVet et al., 2006), sex-specific differences in value distributions could influence reliability if analyzed separately. Accordingly, any apparent bias in reliability is less about differences in central tendency (*e.g.*, mean PPT) between sexes than about within-sex variability, which directly affects metrics such as the ICC, SEM, and MDC (Weir, 2005; DeVet et al., 2006). Future studies should assess whether measurement reliability varies by sex in more balanced samples.

Additionally, the sample comprised participants without musculoskeletal diagnoses, which may be viewed as a limitation. Baseline pain prevalence from the Standardised Nordic Questionnaire differed between groups, and because pain status can influence PPT values and reliability indices, these differences should be considered when interpreting our estimates. Although data collection occurred under conditions comparable to a clinical setting, further confirmation is warranted in clinical populations with diagnosed musculoskeletal conditions.

## Conclusions

This study confirmed that the PPT protocol demonstrates high measurement reliability across body regions, intervention groups, and time points. Its consistency over time and recalculated SEM and MDC values at each follow-up support its suitability for longitudinal applications. These findings position the protocol as a robust tool for evaluating changes in pain sensitivity, particularly in occupational health, with potential applicability to clinical research. Finally, future studies should consider how intervention-related variability influences measurement precision, and researchers should avoid assuming reliability estimates from baseline may be applicable at later time points without reassessment.

## Supplemental Information

10.7717/peerj.20834/supp-1 Supplemental Information 1 Standardized protocol and measurement sheet for pressure pain threshold (PPT) assessment in three anatomical regions The standardized protocol and measurement sheet used for assessing pressure pain threshold (PPT) in the neck (upper trapezius), forearm (extensor carpi ulnaris), and reference region (tibialis anterior). Instructions are available in English, Spanish, and Portuguese to ensure multilingual applicability. The document includes patient information fields, step-by-step instructions for evaluator positioning and pressure application (at 1 kgf/sec), anatomical landmark identification, and a triplicate measurement format for each body region to ensure reliability. This form was used in all measurement sessions by trained physiotherapists in a controlled environment.

10.7717/peerj.20834/supp-2 Supplemental Information 2 Raw pressure pain threshold data, reliability metrics, and codebook for intervention and control groups The raw and processed data related to pressure pain threshold (PPT) assessments across three time points (baseline, 3 months, 6 months) with available-cases to each time point.

10.7717/peerj.20834/supp-3 Supplemental Information 3 Relative and absolute reliability results by Consistency and Complete-case analysis The results regarding relative reliability using the two-way mixed-effects ICC for consistency, alongside absolute indices (SEM, MDC), alongside a table with relative reliability results ICC (absolute agreement) and absolute reliability (SEM, and MDC) using completers only (participants who finished all follow-ups).

10.7717/peerj.20834/supp-4 Supplemental Information 4 STROBE check list for cohort studies Completed STROBE (Strengthening the Reporting of Observational Studies in Epidemiology) checklist for cross-sectional studies, detailing compliance with reporting standards for observational research. The checklist includes references to specific sections of the manuscript where each item is addressed, ensuring transparency and methodological rigor in the reporting of the study.

## Additional Information and Declarations

### Data Availability

The following information was supplied regarding data availability: The raw data of pressure pain threshold measurement is available in the Supplemental Files.
