# Supplementary material for: Reliability of a pressure pain threshold protocol: secondary analysis of a longitudinal trial with cluster randomization
Source: PeerJ. 2026 Feb 25;14:e20834. doi: 10.7717/peerj.20834 (PMC12949580; doi:10.7717/peerj.20834)
Supplement: Supplemental Information 1 — The standardized protocol and measurement sheet used for assessing pressure pain threshold (PPT) in the neck (upper trapezius), forearm (extensor carpi ulnaris), and reference region (tibialis anterior). Instructions are available in English, Spanish, and Portuguese to ensure multilingual applicability. The document includes patient information fields, step-by-step instructions for evaluator positioning and pressure application (at 1 kgf/sec), anatomical landmark identification, and a triplicate measurement format for each body region to ensure reliability. This form was used in all measurement sessions by trained physiotherapists in a controlled environment. [file peerj-14-20834-s001.docx]

**Pressure Pain Threshold (PPT) protocol Aguila-Leppe**

**PPT Measurement Results Sheet**

**English version**

| **Patient Information** | | | | | | | | | |
| --- | --- | --- | --- | --- | --- | --- | --- | --- | --- |
| Name: | | | |  | | | | | |
| Patient ID (optional): | | | |  | | | | | |
| Sex/Gender: | | | |  | | | | | |
| Date of Birth: | | | |  | | | | | |
| Age: | | | |  | | | | | |
| Diagnostic (optional): | | | |  | | | | | |
| **Measurement Information** | | | | | | | |  |  |
| Date: Hour: | | | | | | | |  |  |
| Evaluator Name / ID: | | | | | | | |  |  |
|  | | | | | | |  |  |  |
| **Neck – Upper Trapezius** | | | | | | | |  |  |
| Right Side Measurements | | | | Left Side Measurements | | | Observations |  |  |
| 1st | 2nd | 3rd | | 1st | 2nd | 3rd |  |  |  |
|  |  |  | |  |  |  |  |  |  |
| Mean Right Neck PPT | | | | Mean Left Neck PPT | | |  |  |  |
|  | | | |  | | |  |  |  |
| Neck PPT (Bilateral Mean) | | | | | | |  |  |  |
|  | | | | | | |  |  |  |
|  | | | | | | |  |  |  |
| **Forearm - Extensor Carpi Ulnaris** | | | | | | | |  |  |
| Right Side Measurements | | | | Left Side Measurements | | | Observations |  |  |
| 1st | 2nd | 3rd | | 1st | 2nd | 3rd |  |  |  |
|  |  |  | |  |  |  |  |  |  |
| Mean Right Forearm PPT | | | | Mean Left Forearm PPT | | |  |  |  |
|  | | | |  | | |  |  |  |
| Forearm PPT (Bilateral Mean) | | | | | | |  |  |  |
|  | | | | | | |  |  |  |
|  | | | | | | |  |  |  |
| **Lower Leg – Tibialis Anterior** | | | | | | | |  |  |
| Right Side Measurements | | | | Left Side Measurements | | | Observations |  |  |
| 1st | 2nd | 3rd | | 1st | 2nd | 3rd |  |  |  |
|  |  |  | |  |  |  |  |  |  |
| Mean Right Reference PPT | | | | Mean Left Reference PPT | | |  |  |  |
|  | | | |  | | |  |  |  |
| Reference PPT (Bilateral Mean) | | | | | | |  |  |  |
|  | | | | | | |  |  |  |

**Pressure Pain Threshold (PPT) protocol Aguila-Leppe**

**Measurement Information**

| **Item Check-List:**   - Digital Algometer (charged) - Rigid Measuring Tape - Chronometer | - Pencil - Non-Permanent Marker - Calculator |
| --- | --- |

| **PPT measurement instructions** |
| --- |
| Provide the following instructions to measure the PPT:  *“Today we will perform a pain pressure threshold measurement, in which I will apply pressure with the algometer, and you will tell me when the pressure turns into a painful or unpleasant sensation. The idea is not to endure the pain but to be aware of the moment when the sensation of pain appears while pressing. This sensation should be different from that of pressure, changing from a feeling of pressure to an unpleasant or painful sensation”.* |
| Note: If the participant had any concern about the differences between the words “unpleasant” and “pain”, establish that what is requested is the first sensation that changed from pressure to unpleasant. |

| **Position of the patient and demonstration** | |
| --- | --- |
| Begin by seating the participant on the examination table to deliver instructions and perform the demonstration. Then, instruct them to lie supine, ensuring that clothing does not interfere with the neck, forearm, and lower leg measurement sites. | 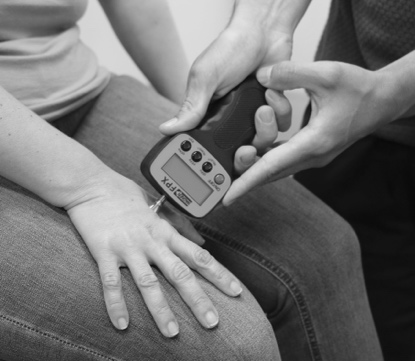 |

| **Rate of measurement** |
| --- |
| Apply a perpendicular force between the rubber tip and the surface marked on each body region. The pressure rate must be maintained at 1 kg/cm^2^/sec until the participant indicates their PPT. Each site should be measured three times, with a 30-second interval between repetitions to minimize temporal summation.  Note: When placing the tip of the device perpendicularly on the skin of the body region, reset the pressure count by clicking on the “zero” button. This will ensure that the pressure starts from zero. |
|  |

| **Body regions** | |
| --- | --- |
|  | |
| **Neck PPT**  Using a rigid measuring tape, locate the upper trapezius by identifying the midpoint between the C7 spinous process and the acromion. Mark the muscle belly at this point for assessment. | 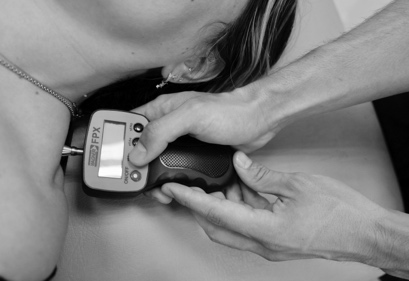 |
|  |  |
| **Forearm PPT**  Identify the extensor carpi ulnaris by measuring 5 cm distal from the lateral epicondyle. Mark the muscle belly and confirm the location by asking the participant to lift the index and middle fingers against resistance. | 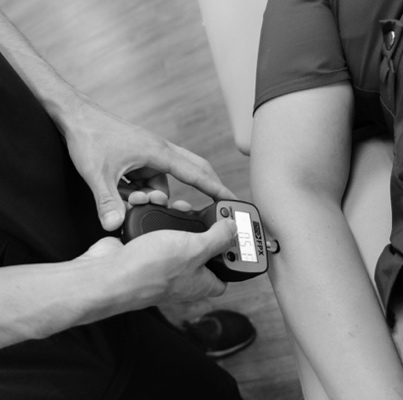 |
|  |  |
| **Lower Leg PPT**  Locate the tibialis anterior muscle belly by measuring 5 cm distal to the fibular head. Mark the site and confirm with dorsiflexion as the participant raises their toes. | 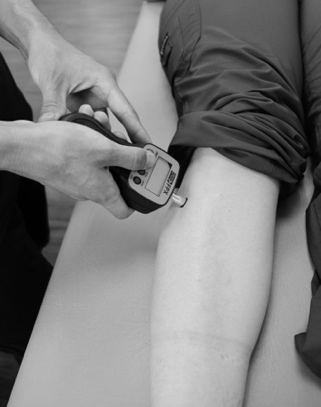 |

**Protocolo de Medición de Umbral de Dolor por Presión (UDP)**

**Hoja de Registro de Medición UDP (kgf)**

**Versión en Español**

| **Información del Paciente** | | | | | | | | | |
| --- | --- | --- | --- | --- | --- | --- | --- | --- | --- |
| Nombre: | | | |  | | | | | |
| ID del paciente (opcional): | | | |  | | | | | |
| Sexo/Género: | | | |  | | | | | |
| Fecha de nacimiento: | | | |  | | | | | |
| Edad: | | | |  | | | | | |
| Diagnóstico (opcional): | | | |  | | | | | |
| **Información de la Medición** | | | | | | | |  |  |
| Fecha: Hora: | | | | | | | |  |  |
| Nombre del Evaluador / ID: | | | | | | | |  |  |
|  | | | | | | |  |  |  |
| **Cuello – Trapecio Superior** | | | | | | | |  |  |
| Mediciones Lado Derecho | | | | Mediciones Lado Izquierdo | | | Observaciones |  |  |
| 1^a^ | 2^a^ | 3^a^ | | 1^a^ | 2^a^ | 3^a^ |  |  |  |
|  |  |  | |  |  |  |  |  |  |
| Promedio Cuello Derecho | | | | Promedio Cuello Izquierdo | | |  |  |  |
|  | | | |  | | |  |  |  |
| UDP Cuello (Promedio ambos lados) | | | | | | |  |  |  |
|  | | | | | | |  |  |  |
|  | | | | | | |  |  |  |
| **Ante Brazo - Extensor Ulnar del Carpo** | | | | | | | |  |  |
| Mediciones Lado Derecho | | | | Mediciones Lado Izquierdo | | | Observaciones |  |  |
| 1^a^ | 2^a^ | 3^a^ | | 1^a^ | 2^a^ | 3^a^ |  |  |  |
|  |  |  | |  |  |  |  |  |  |
| Promedio Ante Brazo Derecho | | | | Promedio Ante Brazo Izquierdo | | |  |  |  |
|  | | | |  | | |  |  |  |
| UDP Ante Brazo (Promedio ambos lados) | | | | | | |  |  |  |
|  | | | | | | |  |  |  |
|  | | | | | | |  |  |  |
| **Pierna – Tibial Anterior** | | | | | | | |  |  |
| Mediciones Lado Derecho | | | | Mediciones Lado Izquierdo | | | Observaciones |  |  |
| 1^a^ | 2^a^ | 3^a^ | | 1^a^ | 2^a^ | 3^a^ |  |  |  |
|  |  |  | |  |  |  |  |  |  |
| Promedio Referencia Derecha | | | | Promedio Referencia Izquierda | | |  |  |  |
|  | | | |  | | |  |  |  |
| UDP Referencia (Promedio ambos lados) | | | | | | |  |  |  |
|  | | | | | | |  |  |  |

**Protocolo de Medición de Umbral de Dolor por Presión (UDP)**

**Instrucciones del Protocolo de Medición PPT**

| **Lista de Instrumentos:**   - Algómetro Digital (cargado) - Cinta Rígida - Cronómetro | - Lapiz - Marcador No Permanente - Calculadora |
| --- | --- |

| **Instrucciones del Protocolo de Medición** |
| --- |
| Proporcione las siguientes instrucciones para medir el UDP:  *“Hoy vamos a realizar la medición del umbral de presión del dolor, en la que aplicaré presión con el algómetro y usted me dirá cuándo la presión se convierte en una sensación dolorosa o desagradable. No se trata de soportar el dolor, sino de ser consciente del momento en que aparece la sensación de dolor al presionar. Esta sensación debe ser diferente de la de una presión, pasando de una sensación de presión a una sensación desagradable o dolorosa”.* |
| Nota: Si el participante muestra alguna inquietud sobre las diferencias entre las palabras «molestia» y «dolor», establezca que lo que se solicita es la primera sensación que pasó de presión a desagradable. |

| **Preparación del participante** | |
| --- | --- |
| Comience sentando al participante en la mesa de exploración para darle las instrucciones y realizar la demostración. A continuación, indíqueles que se coloquen en decúbito supino, asegurándose de que la ropa no interfiera con los puntos de medición del cuello, el antebrazo y la parte inferior de la pierna. | 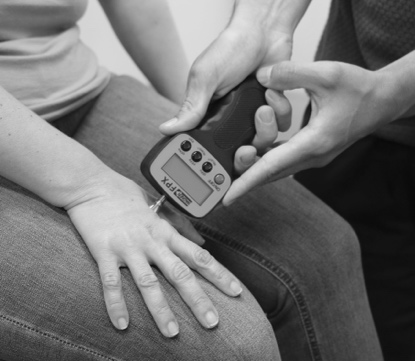 |

| **Tasa de medición** |
| --- |
| Aplique una fuerza perpendicular entre la punta de goma y la superficie marcada en cada región corporal. La tasa de presión debe mantenerse en 1 kg/cm^2^/seg hasta que el participante indique su PPT. Cada sitio debe medirse tres veces, con un intervalo de 30 segundos entre repeticiones para minimizar la suma temporal.  Nota: Al colocar la punta del aparato perpendicularmente sobre la piel de la región corporal, reinicie el recuento de la presión pulsando el botón «cero». Esto asegurará que la presión comience desde cero. |
|  |

| **Zonas Anatómicas y Procedimiento** | |
| --- | --- |
|  | |
| **Cuello**  Con una cinta métrica rígida, localice el trapecio superior identificando el punto medio entre la apófisis espinosa C7 y el acromion. Marque el vientre del músculo en este punto para su evaluación. | 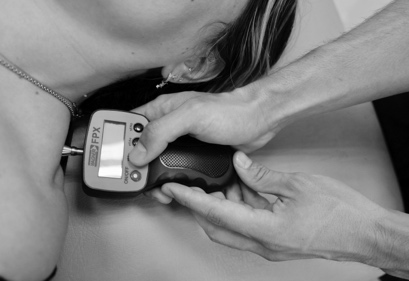 |
|  |  |
| **Ante Brazo**  Identifique el extensor ulnar del carpo midiendo 5 cm a distal del epicóndilo lateral. Marque el vientre del músculo y confirme la localización pidiendo al participante que levante los dedos índice y medio contra resistencia. | 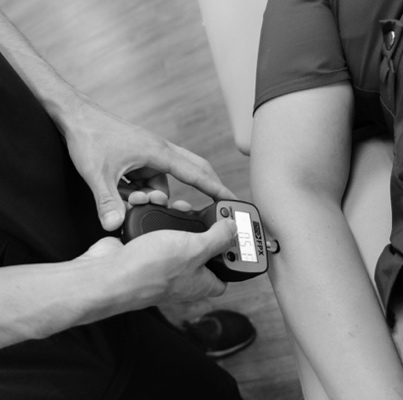 |
|  |  |
| **Pierna**  Localice el vientre del músculo tibial anterior midiendo 5 cm distal a la cabeza de la fíbula. Marque el lugar y confirme con dorsiflexión mientras el participante levanta los dedos de los pies. | 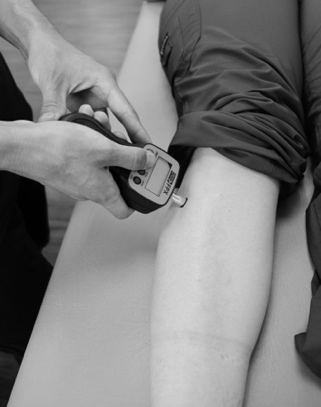 |

**Protocolo de medição do Limiar de Dor por Pressão (LDP)**

**Folha de registro de Medição do LDP (kgf)**

**Versão em Portugués**

| **Informações Sobre o Paciente** | | | | | | | | | |
| --- | --- | --- | --- | --- | --- | --- | --- | --- | --- |
| Nome: | | | |  | | | | | |
| ID do paciente (opcional): | | | |  | | | | | |
| Sexo/ Gênero: | | | |  | | | | | |
| Data de nascimento: | | | |  | | | | | |
| Idade: | | | |  | | | | | |
| Diagnóstico (opcional): | | | |  | | | | | |
| **Informações da Medição** | | | | | | | |  |  |
| Data: Hora: | | | | | | | |  |  |
| Nome do Evaluador / ID: | | | | | | | |  |  |
|  | | | | | | |  |  |  |
| **Pescoço – Trapézio Superior** | | | | | | | |  |  |
| Medições Lado Direito | | | | Medições Lado Esquerdo | | | Observações |  |  |
| 1^a^ | 2^a^ | 3^a^ | | 1^a^ | 2^a^ | 3^a^ |  |  |  |
|  |  |  | |  |  |  |  |  |  |
| Media Pescoço Direito | | | | Media Pescoço Ezquerdo | | |  |  |  |
|  | | | |  | | |  |  |  |
| LDP Pescoço (Média de ambos os lados) | | | | | | |  |  |  |
|  | | | | | | |  |  |  |
|  | | | | | | |  |  |  |
| **Antebraço - Extensor Ulnar do Carpo** | | | | | | | |  |  |
| Medições Lado Direito | | | | Medições Lado Esquerdo | | | Observações |  |  |
| 1^a^ | 2^a^ | 3^a^ | | 1^a^ | 2^a^ | 3^a^ |  |  |  |
|  |  |  | |  |  |  |  |  |  |
| Média do Antebraço Direito | | | | Média do Antebraço Esquerdo | | |  |  |  |
|  | | | |  | | |  |  |  |
| LDP Antebraço (Média de ambos os lados) | | | | | | |  |  |  |
|  | | | | | | |  |  |  |
|  | | | | | | |  |  |  |
| **Perna - Tibial anterior** | | | | | | | |  |  |
| Medições Lado Direito | | | | Medições Lado Esquerdo | | | Observações |  |  |
| 1^a^ | 2^a^ | 3^a^ | | 1^a^ | 2^a^ | 3^a^ |  |  |  |
|  |  |  | |  |  |  |  |  |  |
| Média da Referência Direita | | | | Média da Referência Esquerda | | |  |  |  |
|  | | | |  | | |  |  |  |
| LDP Referência (Média de ambos os lados) | | | | | | |  |  |  |
|  | | | | | | |  |  |  |

**Protocolo de Medição do Limiar de Dor por Pressão (LPT)**

**Instruções do protocolo de medição do LDP**

| **Lista de Instrumentos:**   - Algômetro digital (carregado) - Fita rígida - Cronômetro | - Lápis - Marcador não permanente - Calculadora |
| --- | --- |

| **Instruções para o Protocolo de Medição** |
| --- |
| Apresente as seguintes instruções para medir o PPT:  “Hoje faremos uma medição do limiar de pressão da dor, na qual aplicarei pressão com o algômetro, e você me dirá quando a pressão se transformar em uma sensação dolorosa ou desagradável. A ideia não é suportar a dor, mas estar ciente do momento em que a sensação de dor aparece durante a pressão. Essa sensação deve ser diferente da sensação de pressão, mudando de uma sensação de pressão para uma sensação desagradável ou dolorosa”. |
| Observação: Se o participante tiver alguma preocupação com as diferenças entre as palavras “desagradável” e “dor”, estabeleça que o que está sendo solicitado é a primeira sensação que mudou de pressão para desagradável.. |

| **Preparação dos participantes** | |
| --- | --- |
| Comece sentando o participante na mesa de exame para dar instruções e realizar a demonstração. Em seguida, instrua-o a se deitar em decúbito dorsal, garantindo que a roupa não interfira nos locais de medição do pescoço, antebraço e perna. | 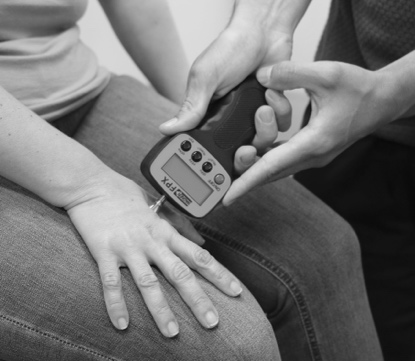 |

| **Taxa de medição** |
| --- |
| Aplique uma força perpendicular entre a ponta de borracha e a superfície marcada em cada região do corpo. A taxa de pressão deve ser mantida em 1 kg/cm^2^/segundo até que o participante indique sua PPT. Cada local deve ser medido três vezes, com um intervalo de 30 segundos entre as repetições para minimizar a soma temporal.  Observação: Ao colocar a ponta do dispositivo perpendicularmente sobre a pele da região do corpo, reinicie a contagem de pressão clicando no botão “zero”. Isso garantirá que a pressão comece do zero. |
|  |

| **Regiões do corpo** | |
| --- | --- |
|  | |
| **Pescoço**  Usando uma fita métrica rígida, localize o trapézio superior identificando o ponto médio entre o processo espinhoso de C7 e o acrômio. Marque o ventre muscular nesse ponto para avaliação. | 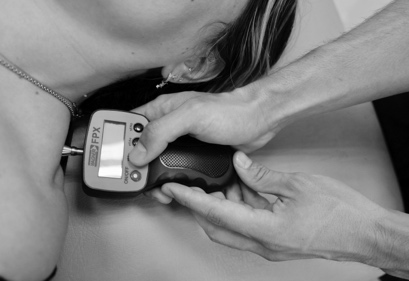 |
|  |  |
| **Antebraço**  Identifique o extensor ulnar do carpo medindo 5 cm distal ao epicôndilo lateral. Marque o ventre do músculo e confirme a localização pedindo ao participante que levante os dedos indicador e médio contra resistência. | 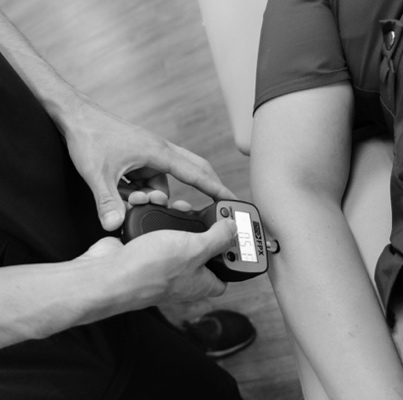 |
|  |  |
| **Perna**  Encontre o ventre do músculo tibial anterior medindo 5 cm distal à cabeça da fíbula. Marque o local e confirme com dorsiflexão enquanto o participante levanta os dedos dos pés. | 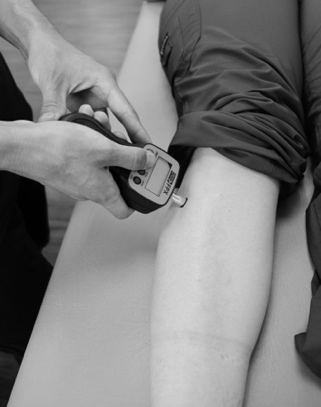 |
