# Supplementary material for: Reliability of a pressure pain threshold protocol: secondary analysis of a longitudinal trial with cluster randomization
Source: PeerJ. 2026 Feb 25;14:e20834. doi: 10.7717/peerj.20834 (PMC12949580; doi:10.7717/peerj.20834)
Supplement: Supplemental Information 3 — The results regarding relative reliability using the two-way mixed-effects ICC for consistency, alongside absolute indices (SEM, MDC), alongside a table with relative reliability results ICC (absolute agreement) and absolute reliability (SEM, and MDC) using completers only (participants who finished all follow-ups). [file peerj-14-20834-s003.docx]

**Supplementary Material 3**

| Relative and Absolute Reliability by Group, Body Region, and Time Points for Available-cases (Consistency-based) | | | | | | | | | | |
| --- | --- | --- | --- | --- | --- | --- | --- | --- | --- | --- |
|  |  | Baseline Active (n = 60) Control (n = 42) | | | 3 Months Active (n = 55) Control (n = 38) | | | 6 Months Active (n = 52) Control (n = 37) | | |
| Body  Region | Group | ICC  (95% CI) | SEM | MDC | ICC  (95% CI) | SEM | MDC | ICC  (95% CI) | SEM | MDC |
| Neck | Active | 0.91  (0.85 - 0.95) | 0.27 | 0.63 | 0.86  (0.76 - 0.92) | 0.36 | 0.84 | 0.85  (0.73 - 0.91) | 0.32 | 0.73 |
|  | Control | 0.94  (0.89 - 0.97) | 0.25 | 0.59 | 0.93  (0.87 - 0.97) | 0.24 | 0.55 | 0.93  (0.87 - 0.97) | 0.24 | 0.56 |
|  | Total  (n = 102) | 0.93  (0.90 - 0.95) | 0.26 | 0.61 | - | - | - | - | - | - |
| Forearm | Active | 0.91  (0.86 - 0.95) | 0.29 | 0.67 | 0.90  (0.84 - 0.94) | 0.28 | 0.65 | 0.91  (0.84 - 0.95) | 0.24 | 0.55 |
|  | Control | 0.91  (0.84 - 0.95) | 0.35 | 0.81 | 0.92  (0.85 - 0.96) | 0.23 | 0.53 | 0.93  (0.87 - 0.97) | 0.21 | 0.49 |
|  | Total  (n = 102) | 0.91  (0.87 - 0.94) | 0.32 | 0.73 | - | - | - | - | - | - |
| Reference | Active | 0.92  (0.86 - 0.95) | 0.42 | 0.97 | 0.93  (0.88 - 0.96) | 0.42 | 0.96 | 0.86  (0.75 - 0.92) | 0.51 | 1.19 |
|  | Control | 0.94  (0.88 - 0.97) | 0.48 | 1.12 | 0.92  (0.84 - 0.96) | 0.44 | 1.03 | 0.95  (0.90 - 0.97) | 0.36 | 0.83 |
|  | Total  (n = 102) | 0.92  (0.86 - 0.95) | 0.48 | 1.1 | - | - | - | - | - | - |
| ICC: Intraclass Correlation Coefficient, SEM: Standard Error Measurement, MDC: Minimal Detectable Change (90%) | | | | | | | | | | |

| Relative and Absolute Reliability by Group, Body Region, and Time Points (Complete-cases) | | | | | | | | | | |
| --- | --- | --- | --- | --- | --- | --- | --- | --- | --- | --- |
|  |  | Baseline | | | 3 Months | | | 6 Months | | |
| Body  Region | Group | ICC  (95% CI) | SEM | MDC | ICC  (95% CI) | SEM | MDC | ICC  (95% CI) | SEM | MDC |
| Neck | Active  (n = 51) | 0.90  (0.81 - 0.95) | 0.30 | 0.69 | 0.85  (0.74 - 0.91) | 0.38 | 0.88 | 0.84  (0.73 - 0.91) | 0.32 | 0.74 |
|  | Control  (n = 37) | 0.94  (0.88 - 0.97) | 0.26 | 0.60 | 0.93  (0.87 - 0.97) | 0.24 | 0.57 | 0.93  (0.87 - 0.97) | 0.24 | 0.56 |
|  | Total  (n = 88) | 0.92  (0.87 - 0.95) | 0.28 | 0.65 | - | - | - | - | - | - |
| Forearm | Active  (n = 51) | 0.91  (0.85 - 0.95) | 0.30 | 0.69 | 0.90  (0.82 - 0.94) | 0.29 | 0.67 | 0.91  (0.83 - 0.95) | 0.24 | 0.56 |
|  | Control  (n = 37) | 0.88  (0.77 - 0.94) | 0.37 | 0.85 | 0.92  (0.85 - 0.96) | 0.23 | 0.53 | 0.93  (0.85 - 0.96) | 0.22 | 0.52 |
|  | Total  (n = 88) | 0.90  (0.85 - 0.93) | 0.32 | 0.73 | - | - | - | - | - | - |
| Reference | Active  (n = 51) | 0.89  (0.74 - 0.95) | 0.49 | 1.10 | 0.93  (0.87 - 0.96) | 0.43 | 0.99 | 0.86  (0.76 - 0.92) | 0.51 | 1.19 |
|  | Control  (n = 37) | 0.94  (0.89 - 0.97) | 0.44 | 1.00 | 0.92  (0.85 - 0.96) | 0.43 | 1.00 | 0.95  (0.90 - 0.97) | 0.36 | 0.83 |
|  | Total  (n = 88) | 0.92  (0.86 - 0.95) | 0.47 | 1.10 | - | - | - | - | - | - |
| ICC: Intraclass Correlation Coefficient, SEM: Standard Error Measurement, MDC: Minimal Detectable Change (90%) | | | | | | | | | | |
