# Supplementary material for: Reliability of a pressure pain threshold protocol: secondary analysis of a longitudinal trial with cluster randomization
Source: PeerJ. 2026 Feb 25;14:e20834. doi: 10.7717/peerj.20834 (PMC12949580; doi:10.7717/peerj.20834)
Supplement: Supplemental Information 4 — Completed STROBE (Strengthening the Reporting of Observational Studies in Epidemiology) checklist for cross-sectional studies, detailing compliance with reporting standards for observational research. The checklist includes references to specific sections of the manuscript where each item is addressed, ensuring transparency and methodological rigor in the reporting of the study. [file peerj-14-20834-s004.doc]

STROBE Statement—Checklist of items that should be included in reports of ***cohort studies***

|  | Item No | Recommendation | In our paper |
| --- | --- | --- | --- |
| **Title and abstract** | 1 | (*a*) Indicate the study’s design with a commonly used term in the title or the abstract | Page 1, line 1-2 |
|  |  | (*b*) Provide in the abstract an informative and balanced summary of what was done and what was found | Page 1, lines 13-30 |
| Introduction | | |  |
| Background/rationale | 2 | Explain the scientific background and rationale for the investigation being reported | Page 2, lines 33-63 provide the background  Page 2, lines 64-80 explain the rationale |
| Objectives | 3 | State specific objectives, including any prespecified hypotheses | Page 3, lines 81-85 |
| Methods | | |  |
| Study design | 4 | Present key elements of study design early in the paper | Page 3, lines 89-91 |
| Setting | 5 | Describe the setting, locations, and relevant dates, including periods of recruitment, exposure, follow-up, and data collection | Page 3, lines 99-101 |
| Participants | 6 | (*a*) Give the eligibility criteria, and the sources and methods of selection of participants. Describe methods of follow-up | Page 3, lines 101-105 |
|  |  | (*b*) For matched studies, give matching criteria and number of exposed and unexposed | Not applicable |
| Variables | 7 | Clearly define all outcomes, exposures, predictors, potential confounders, and effect modifiers. Give diagnostic criteria, if applicable | Page 3, lines 108-115 define the main outcome  Page 3 lines 91-97 provides the exposure to the interventions.  No predictors, potential confounders, effect modifiers and diagnostic criteria were considered. |
| Data sources/ measurement | 8* | For each variable of interest, give sources of data and details of methods of assessment (measurement). Describe comparability of assessment methods if there is more than one group | Page 3, line 89 provide the source of the data  Pages 3-4, lines 116-165 explains the methods of measurement. |
| Bias | 9 | Describe any efforts to address potential sources of bias | - |
| Study size | 10 | Explain how the study size was arrived at | - |
| Quantitative variables | 11 | Explain how quantitative variables were handled in the analyses. If applicable, describe which groupings were chosen and why | Page 5, lines 176-183 explain how quantitative variables were handled in the analyses |
| Statistical methods | 12 | (*a*) Describe all statistical methods, including those used to control for confounding | Page 5, lines 176-191 |
|  |  | (*b*) Describe any methods used to examine subgroups and interactions | Not applicable |
|  |  | (*c*) Explain how missing data were addressed | Page 5, lines 194-195 |
|  |  | (*d*) If applicable, explain how loss to follow-up was addressed | Page 5, lines 194-195 |
|  |  | (*e*) Describe any sensitivity analyses | Not applicable |
| Results | | |  |
| Participants | 13* | (a) Report numbers of individuals at each stage of study—eg numbers potentially eligible, examined for eligibility, confirmed eligible, included in the study, completing follow-up, and analysed | Page 6, line 198-200 provides de number of individuals included in the study, those who completed follow-up and those analysed. Figure 1. |
|  |  | (b) Give reasons for non-participation at each stage | - |
|  |  | (c) Consider use of a flow diagram | - |
| Descriptive data | 14* | (a) Give characteristics of study participants (eg demographic, clinical, social) and information on exposures and potential confounders | Page 6, lines 200-203. Table 1 |
|  |  | (b) Indicate number of participants with missing data for each variable of interest | - |
|  |  | (c) Summarise follow-up time (eg, average and total amount) | - |
| Outcome data | 15* | Report numbers of outcome events or summary measures over time | Page 6, lines 208-215 provides summary of measurements over time. Table 2 |
| Main results | 16 | (*a*) Give unadjusted estimates and, if applicable, confounder-adjusted estimates and their precision (eg, 95% confidence interval). Make clear which confounders were adjusted for and why they were included | Page 6, lines 219-236 provides unadjusted estimates with their precision. Table 3 |
|  |  | (*b*) Report category boundaries when continuous variables were categorized | Not Applicable |
|  |  | (*c*) If relevant, consider translating estimates of relative risk into absolute risk for a meaningful time period | Not applicable |
| Other analyses | 17 | Report other analyses done—eg analyses of subgroups and interactions, and sensitivity analyses | Not Applicable |
| Discussion | | |  |
| Key results | 18 | Summarise key results with reference to study objectives | Page 7-8, lines 240-296 |
| Limitations | 19 | Discuss limitations of the study, taking into account sources of potential bias or imprecision. Discuss both direction and magnitude of any potential bias | Page 8, lines 297-303 |
| Interpretation | 20 | Give a cautious overall interpretation of results considering objectives, limitations, multiplicity of analyses, results from similar studies, and other relevant evidence | Page 8, lines 304-311 |
| Generalisability | 21 | Discuss the generalisability (external validity) of the study results | Page 8, lines 304-311 |
| Other information | | |  |
| Funding | 22 | Give the source of funding and the role of the funders for the present study and, if applicable, for the original study on which the present article is based | ‘Funding’ section |

*Give information separately for exposed and unexposed groups.

**Note:** An Explanation and Elaboration article discusses each checklist item and gives methodological background and published examples of transparent reporting. The STROBE checklist is best used in conjunction with this article (freely available on the Web sites of PLoS Medicine at http://www.plosmedicine.org/, Annals of Internal Medicine at http://www.annals.org/, and Epidemiology at http://www.epidem.com/). Information on the STROBE Initiative is available at http://www.strobe-statement.org.
